# Editorial: Function and regulation of non-neuronal cells in the nervous system

**DOI:** 10.3389/fncel.2025.1550903

**Published:** 2025-02-07

**Authors:** Sisi Li, Yin Cai, Zhengyuan Xia

**Affiliations:** ^1^Department of Anesthesiology, Stomatological Hospital of Chongqing Medical University, Chongqing, China; ^2^Department of Health Technology and Informatics, The Hong Kong Polytechnic University, Hong Kong, Hong Kong SAR, China; ^3^Department of Anesthesiology and Perioperative Medicine, Zhengzhou Central Hospital Affiliated to Zhengzhou University, Zhengzhou, China; ^4^State Key Laboratory of Pharmaceutical Biotechnology, Department of Medicine, The University of Hong Kong, Hong Kong, Hong Kong SAR, China; ^5^Doctoral Training Platform for Research and Translation, Shuanghe, Hubei, China

**Keywords:** non-neuronal cells, nervous system, neuroimmunology, neuroinflammation, neurovascular coupling

## Introduction

Traditionally, the nervous system has been perceived primarily as a complex network predominantly composed of neurons. Nevertheless, ongoing developments in the field of neuroscience have brought to light the significant contributions of non-neuronal cells, highlighting their importance (Kruyer, 2024; Cahill et al., 2024). Getting a profound insight into their functional traits and the mechanisms that govern them is essential for developing innovative approaches to treating and preventing neurological disorders.

Recently, there has been significant progress in the investigation of non-neuronal cells within the nervous system. The primary emphasis lies in uncovering the intricate network of interactions that connect these cells to neurons and to each other. As an illustration, recent studies in the field of neurovascular coupling have begun to elucidate the intricate relationship between brain activity and the modulation of blood flow within the cerebral system (Chen et al., 2024; Gordon, 2024; Tournissac et al., 2024; Omura et al., 2024). Investigations into neuroimmunology have introduced novel insights into the critical roles that immune cells play in both inflammatory response in the nervous system and the progression of neurodegenerative disorders (Ortega et al., 2024; Heneka et al., 2024). Investigating the interactions between glial cells serves as a burgeoning area of study, focusing on unraveling the complexities of communication and the exchange of information among astrocytes, other glial cells, and neurons, along with their significant influence on neural operations (Cahill et al., 2024; Sun et al., 2024; de Ceglia et al., 2023).

## The pivotal importance of non-neuronal cells within the nervous system

Microglia, the primary resident immune cells found within the nervous system, act as the initial barrier against invasive pathogens and play an essential role in upholding the immune balance of the neural environment (Pereira-Iglesias et al., 2025). These cells play a significant part in essential processes such as the elimination of excess synapses throughout neural maturation and are capable of rapidly triggering protective immune mechanisms when faced with injury or pathological conditions in the nervous system (Hu and Tao, 2024; Niiyama et al., 2023; Lehrman et al., 2018). Astrocytes play a vital role not only in offering critical structural support for neurons but also in expertly managing the intricate balance of neurotransmitter absorption and release (Santello and Volterra, 2009; Agulhon et al., 2010), meticulously maintaining ion equilibrium (Walz, 2000; Gabriel et al., 2004), and influencing synaptic adaptability (Murphy-Royal et al., 2020). Cells within the neurovascular unit, including the endothelial and pericyte populations, are essential for maintaining the stability and integrity of the blood-brain barrier while ensuring the accurate and suitable regulation of cerebral perfusion (Zhang et al., 2024; Webb et al., 2024).

In the field of neurological disorders, the unnormal functioning of glial cells is commonly identified. In conditions such as Alzheimer's and Parkinson's diseases, the involvement of immune cells and astrocytes in the inflammatory response has become a characteristic feature of these disorders (Rojo and Gibson, 2023; Serrano-Pozo et al., 2024; Roodveldt et al., 2024). Additionally, impairments in the mechanisms that link neural function with vascular responses and disruptions to the integrity of the neural barrier are also critical factors in the development of numerous neurological disorders, including stroke and multiple sclerosis (Srinivasan et al., 2011; Spencer et al., 2018; Lopes Pinheiro et al., 2016). Consequently, a thorough exploration of the functions and mechanisms of non-neuronal cells throughout the disease progression is anticipated to yield novel perspectives and strategic avenues for identifying new therapeutic targets.

## This Research Topic centers around a specific range of topics

This Research Topic provides an examination of important aspects regarding non-neuronal cells within the nervous system.

Non-neuronal cells (mainly glial cells) play roles in supporting, protecting, and nourishing neurons in the nervous system. Their ability to divide makes them prone to mutation and malignant transformation. Most of malignant tumors in the central nervous system originate from non-neuronal cells. Ji et al. reported the discovery of Gap Junction Protein, Gamma 1(GJC1) as a prognostic biomarker in glioma cells. GJC1 is located on human chromosome 17 and encodes the gap junction gamma–1 protein (connexin 45, Cx45), which participates in intercellular communication. The expression of Cx45 is decreased in colorectal cancer and has a tumor-suppressive role in melanoma cells, but its function in gliomas remains unclear. The study of Ji et al. systematically investigated the influence of clinicopathological features, molecular subclasses, and prognosis of gliomas on GJC1 expression patterns. They analyzed the biological processes and markers associated with GJC1 in tumor cells and further performed drug correlation analysis. Moreover, all the specific mechanisms of drug action obtained from the drug correlation analysis were related to the cell cycle, further supporting the influence of GJC1 on cell-cycle regulation.

The review article of Zhao et al. comprehensively stated olfactory system's complexity and the pivotal roles glial cells play in both health and disease conditions. This review discussed the diverse functions and dynamics of glial cells in the mammalian olfactory bulb, mainly focused on astrocytes, microglia, oligodendrocytes, olfactory ensheathing cells, and radial glia cells. Each type of glial contributes uniquely to the olfactory bulb's functionality, influencing many processes from synaptic modulation and neuronal survival to immune defense and axonal guidance. The review features their roles in maintaining neural health, their involvement in neurodegenerative diseases, and their potential therapeutic applications for neuroregeneration.

Traumatic brain injury (TBI) is a critical global health concern characterized by elevated rates of both morbidity and mortality. The pathological and physiological changes after TBI are closely related to microglia. Microglia, the primary immune cells in the brain, are closely linked to the mechanisms and treatment of TBI. Zhang et al. published a bibliometric analysis and visualization study to identify current research hotspots and predict future. In this study, the authors meticulously discussed the mechanism of action of non-neuronal cells in ischemic stroke from two aspects: the repair of the blood-brain barrier and the immune infiltration following TBI and post- TBI peripheral immunosuppression and inflammation.

Ischemic stroke accounts for 75% to 80% of all stroke events, making it the leading cause of cerebrovascular diseases and related deaths worldwide (Alonso et al., 2019). Following ischemic stroke, non-neuronal cells within the nervous system play a crucial role in maintaining neurovascular unit functions, regulating metabolic and inflammatory processes of the nervous system. Wang et al. systemically explored the global research trends and prospects of immune-related therapy in ischemic stroke.

To summarize, the field of research focused on non-neuronal cells within the nervous system is currently experiencing a significant growth. This dedicated Research Topic is intended to significantly support the advancement of this area by establishing a platform for academic discussions and for the demonstration of research achievements.
